# Draft genome sequence of the oomycete *Pythium destruens* strain ATCC 64221 from a horse with pythiosis in Australia

**DOI:** 10.1186/s13104-020-05168-1

**Published:** 2020-07-09

**Authors:** Theerapong Krajaejun, Weerayuth Kittichotirat, Preecha Patumcharoenpol, Thidarat Rujirawat, Tassanee Lohnoo, Wanta Yingyong

**Affiliations:** 1 Department of Pathology, Faculty of Medicine, Ramathibodi Hospital, Mahidol University, Bangkok, Thailand; 2 Systems Biology and Bioinformatics Research Group, Pilot Plant Development and Training Institute, King Mongkut’s University of Technology Thonburi, Bangkhuntien, Bangkok, Thailand; 3 Research Center, Faculty of Medicine, Ramathibodi Hospital, Mahidol University, Bangkok, Thailand

**Keywords:** *Pythium destruens*, *Pythium insidiosum*, Pythiosis, Genome sequence, Australia

## Abstract

**Objectives:**

Genome sequences are a vital resource for accelerating the biological exploration of an organism of interest. *Pythium destruens* (a synonym of *Pythium insidiosum*) causes a difficult-to-treat infectious disease called pythiosis worldwide. Detection and management of pythiosis are challenging. Basic knowledge of the disease is lacking. Genomes of this organism isolated from different continents (i.e., Asia and the Americas) have been sequenced and publicly available. Here, we sequenced the genome of an Australian isolate of *P. destruens*. Genome data will facilitate the comparative analysis of this and related species at the molecular level.

**Data description:**

Genomic DNA of the *P. destruens* strain ATCC 64221, isolated from a horse with pythiosis in Australia, was used to prepare one paired-end library (with 180-bp insert) for next-generation sequencing, using the Illumina HiSeq 2500 short-read platform. Raw reads were cleaned and assembled by several bioinformatics tools. A total of 20,860,454 processed reads, accounted for 2,614,890,553 total bases, can be assembled into a 37.8-Mb genome, consisting 13,060 contigs (average length: 2896 bases; range: 300–142,967), *N*_50_ of 11,370 bases, and 2.9% ‘N’ composition. The genome was determined 85.9% completeness, contained 14,424 predicted genes, and can be retrieved online at the NCBI/DDBJ databases under the accession number BCFQ01000000.1.

## Objective

Genome sequences of many microorganisms can now be generated at a much lower cost than ever in the past due to the advances in the massive DNA sequencing technology, so-called next-generation sequencing (NGS) platform [1]. Such data are a vital resource for accelerating the exploration of biology and pathogenicity of an organism of interest. The oomycete microorganism *Pythium insidiosum* has emerged as a devastating pathogen for a few decades [2–4]. It is a causative agent of a difficult-to-treat infectious disease called pythiosis, reported in humans and some animals from tropical, subtropical, and temperate areas across the world. Detection and management of patients with pythiosis are complicated and problematic in the clinics due to the lack of efficient diagnostic and therapeutic tools, as well as basic knowledge of the disease. Genomes of 6 *P. insidiosum* strains isolated from different sources (i.e., human, horse, and water) and geographic locations in the continents of Asia and Americas (i.e., the United States, Costa Rica, Brazil, and Thailand) were sequenced and deposited in the public data repositories [5–10], and become an invaluable resource for bioinformatics and functional genetic studies of this organism.

Here, we sequenced a draft genome of *Pythium destruens*, isolated from an equine patient with pythiosis, using the Illumina HiSeq 2500-based NGS platform. The species name of *P. destruens* has been first introduced in 1987 and appears to be a synonym of *P. insidiosum* based on antigenic and phylogenetic analyses [11–13]. The genomic data of *P. destruens* represent a pathogen strain from the continent of Australia. Bioinformatics and comparative genomics analyses of the pathogen genome data reported by this and other studies [5–10] could provide insights into basic biology, genetic variation, host specificity, and underlying pathogenesis mechanism and lead to identifying potential target genes for the development of a novel control measure (i.e., drug and vaccine) against pythiosis.

## Data description

The *P. destruens* strain ATCC 64221 was isolated from a horse with pythiosis in Australia. Its molecular identity information, i.e., ribosomal deoxyribonucleic acid (rDNA) sequence, was stored in the National Center for Biotechnology Information (NCBI) database (accession numbers: KP780446.1 and KP780468.1). The organism was grown on Sabouraud dextrose (SD) agar and regularly subcultured every 3–4 weeks until use. Several small pieces of SD agar containing an actively-growing colony were transferred to SD broth and shaking incubated at 37 °C for 7 days. The well-grown organism was collected from the broth culture and proceeded for genomic deoxyribonucleic acid (gDNA) extraction, following the protocol described by Lohnoo et al. [14]. The organism was re-checked its identity and genotype (clade-II) by the rDNA single-nucleotide polymorphism-based multiplex polymerase chain reaction [13, 15]. The resulting gDNA was then used to prepare one paired-end library (with 180-bp insert) for NGS, using the Illumina HiSeq2500 platform (Yourgene Bioscience, Taiwan). Before genome assembly, the Qiagen CLC Genomics Workbench software was used to trim obtained raw reads to recruit a read length of 35 bases or more. The adaptor sequences of all reads were eliminated by the Cutadapt 1.8.1 program [16]. After sequence trims, a total of 20,860,454 raw reads (average length: 125 bases) were obtained, which accounted for 2,614,890,553 total bases. The Velvet 1.2.10 program [17] can assemble the recruited raw reads into 13,060 contigs with an average length of 2896 bases (range: 300–142,967). The program also reported *N*_50_ of 11,370 bases and percent ‘N’ (unknown bases) of 2.9%. The resulting draft genome of *P. destruens* contained 37,817,292 bases (69× genome coverage). A BLAST search of a CEGMA panel of 248 highly-conserved eukaryotic genes against the assembled sequences showed 85.9% genome completeness [18]. The MAKER2 program [19] predicted 14,424 open reading frames (ORFs). All contig sequences can be downloaded online at the NCBI and DNA Data Bank of Japan (DDBJ) data repositories under the accession number BCFQ01000000.1 (Data file 1; Table 1).

**Table 1:** Overview of data files/data sets

Label	Name of data file/data set	File types (file extension)	Data repository and identifier (DOI or accession number)
Data file 1	*Pythium insidiosum* strain ATCC 64221, whole genome shotgun sequencing project	FASTA	GenBank (https://identifiers.org/ncbi/insdc:BCFQ00000000.1)

In summary, the pathogenic oomycete *P. destruens* (an alternative name or synonym of *P. insidiosum*) can cause a deadly infectious condition “pythiosis” in humans and some animals, especially horses and dogs, worldwide [2, 3, 11–13, 20]. Although some established diagnostic and therapeutic modalities are available, the management of the infection caused by this microorganism is still challenging [20–25]. Little is known regarding the basic biology and pathogenesis of the pathogen. We reported a draft genome sequence of the *P. destruens* strain ATCC 64221, isolated from an infected horse in Australia. The genome was 37.8 Mb in size and comprised of 13,060 contigs, and 14,424 predicted ORFs (which was similar to the ORF number (n = 14,962) predicted in the reference genome from the co-species *P. insidiosum* strain Pi-S [7]). The genome sequence obtained from the current study will serve as an invaluable resource to facilitate comparative genomic and molecular genetic analyses of *P. destruens* and related species, as well as to identify potential target genes for the development of drug and vaccine against pythiosis.

## Limitations

1. The Illumina HiSeq 2500 short-read NGS platform was employed in the genome sequencing of the *P. destruens* strain ATCC 64221. Such a platform relies on DNA amplification for library construction where sequence coverage biases may occur. Besides, the sequencing-by-synthesis technique employed by Illumina platform is known to produce a small number of substitution errors.
2. The draft genome of *P. destruens* was sequenced from a single 180 bp-insert paired-end library, with no mate-pair library. This limitation resulted in a less complete genome with a relatively higher number of assembled sequence fragments (13,060 contigs) and relatively lower genome size (37.8 Mb), in comparison with the draft genome of the *P. insidiosum* strain Pi-S (number of contigs: 1192; genome size: 53.2 Mb) that employed several paired-end (180-bp insert) and mate-pair (insets ranging from 5 to 15 kb) libraries [7]. Comparative analysis of these 2 genomes, for example, to investigate gene gain, loss, and modification is cautioned with such limitations.
3. The mitochondrial genome data were not separated from the nuclear genome data, and may slightly impact the estimated genome size and gene contents of *P. destruens*.

## Data Availability

The draft genome sequence comprising 13,060 contigs (accession numbers BCFQ01000001-BCFQ01013060), is available in GenBank here: https://identifiers.org/ncbi/insdc:BCFQ00000000.1 [26].

## Abbreviations

DNA: Deoxyribonucleic acid
DDBJ: DNA Data Bank of Japan
gDNA: Genomic deoxyribonucleic acid
NCBI: National Center for Biotechnology Information
NGS: Next-generation sequencing
ORF: Open reading frame
rDNA: Ribosomal deoxyribonucleic acid
SD: Sabouraud dextrose
